# Regenerative Therapies Using Cell Sheet-Based Tissue Engineering for Cardiac Disease

**DOI:** 10.4061/2011/845170

**Published:** 2011-10-06

**Authors:** Yuji Haraguchi, Tatsuya Shimizu, Masayuki Yamato, Teruo Okano

**Affiliations:** Institute of Advanced Biomedical Engineering and Science, TWIns, Tokyo Women's Medical University, 8-1 Kawada-cho, Shinjuku-ku, Tokyo 162-8666, Japan

## Abstract

At present, cardiac diseases are a major cause of morbidity and mortality in the world. Recently, a cell-based regenerative medicine has appeared as one of the most potential and promising therapies for improving cardiac diseases. As a new generational cell-based regenerative therapy, tissue engineering is focused. Our laboratory has originally developed cell sheet-based scaffold-free tissue engineering. Three-dimensional myocardial tissue fabricated by stacking cardiomyocyte sheets, which are tightly interconnected to each other through gap junctions, beats simultaneously and macroscopically and shows the characteristic structures of native heart tissue. Cell sheet-based therapy cures the damaged heart function of animal models and is clinically applied. Cell sheet-based tissue engineering has a promising and enormous potential in myocardial tissue regenerative medicine and will cure many patients suffering from severe cardiac disease. This paper summarizes cell sheet-based tissue engineering and its satisfactory therapeutic effects on cardiac disease.

## 1. Introduction

Various clinical therapies including drug-based, catheter-based, surgical-based, and medical device-based therapies for cardiac disease are performed and found to elongate the life-span of patients who suffer cardiac disease. However, cardiac disease still remains a major cause of morbidity and mortality in the world, especially in developed countries [1–3]. Some conventional therapies have several problems, for example, the possible risks of side effects, the requirements of special techniques and repeating therapy, immune rejection, donor shortage, infection, and thrombus, and so forth. Therefore, at present, many researchers in various fields including surgery, internal medicine, pharmacology, medical device technology, chemistry, and cell biology, are actively attempting to find possible solutions for the problems and establish new therapies for curing severe cardiac diseases. 

Cell-based regenerative therapy currently emerges as one of the most promising methods for treating cardiac disease. Regenerative therapy by the direct injection of dissociated cells has been clinically performed, and the modest therapeutic efficacies are confirmed [4–10]. Previous studies in animal models and clinical trials show that many injected cells die after the transplantation and only few transplanted cells are detected in the infarcted myocardium [11, 12]. The poor survival of injected cells hinders more effective therapeutic effects. In addition, the controls of the shape, size, and location of injected cells are difficult in the case of dissociated cell injection. To overcome these problems, tissue engineering is viewed as a new generational cell therapy for cardiac disease [13]. Tissue engineering is currently based on concepts that three-dimensional (3D) scaffolds are used as an alternative for extracellular matrix (ECM), and cells are seeded into the scaffolds [14]. The transplantation of engineered myocardial tissue grafts improves damaged heart functions in animal models, and clinical trials have already started [15–18]. 

On the other hand, our laboratory originally develops cell sheet-based scaffold-free tissue engineering by using a unique culture surface grafted with a temperature-responsive polymer, poly(*N*-isopropylacrylamide) (PIPAAm), that can control the attachment and detachment of live cultured cells by simple temperature changes [19, 20]. This review discusses (1) cell sheet-based scaffold-free tissue engineering, (2) the characters of 3D tissue fabricated by cell sheet engineering, and (3) the therapeutic effects of the tissue for cardiac disease. 

## 2. Preparation of Cell Sheets by Using a Temperature-Responsive Surface

**Figure 1:**
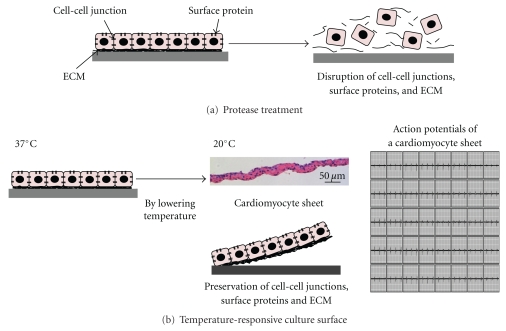
The preparation of a cardiomyocyte sheet using a temperature-responsive culture dish without protease treatment. Typical cell harvest using proteases results in the disruption of cell-cell junctions including gap junctions, cell surface proteins including gap junction precursors, and extracellular matrix (ECM) (a). When temperature-responsive culture surfaces are used, the structures of cells are preserved and cultured cardiomyocytes are released as a contiguous cell sheet (b). A microphotograph shows the cross-sectional view of a cardiomyocyte sheet. Electrograms show the spontaneous action potentials of a cardiomyocyte sheet.

**Figure 2:**
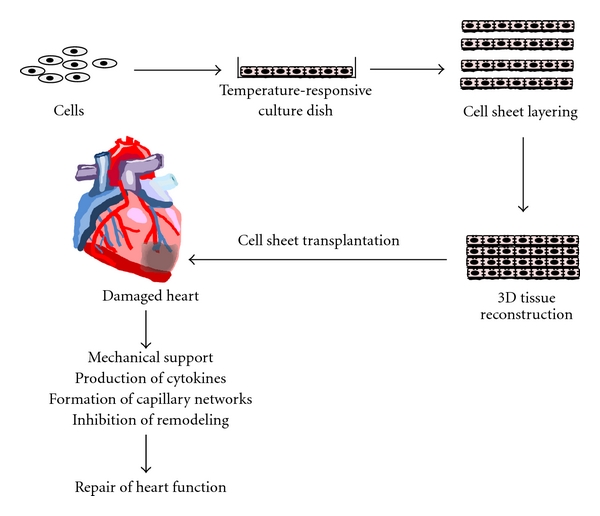
Scaffold-free cell sheet-based tissue engineering: the fabrication of 3D myocardial tissue, transplantation, and the therapeutic effects.

In our laboratory, a unique cell culture surface, which is covalently grafted with a temperature-responsive polymer, PIPAAm, is originally developed [19, 20]. The surfaces are slightly hydrophobic, and cells can adhere and proliferate at 37°C. The hydrophobic surface becomes hydrophilic by lowering temperature below 32°C, and cells are unable to adhere to the surface. The unique surface change allows cultured cells to detach themselves spontaneously from the culture surfaces simply by reducing temperature without any proteolytic treatment [21, 22]. When cells are cultured confluently on the surface, the cells can detach themselves as a contiguous cell sheet without the disruption of cell-cell junctions by reducing culture temperature (Figure 1) [21, 22]. In addition to cell-cell junctions, fibronectin matrix, which is a major ECM component mediating cell adhesion onto culture dishes, on cell sheets is preserved even after their detachment (Figure 1) [21–23]. Due to the presence of deposited ECM produced during cultivation, cell sheets can be easily attached to other surfaces such as culture dishes, other cell sheets, and even host tissues. Therefore, 3D tissues can be easily created by layering cell sheets without scaffolds (Figure 2). Cell sheet-based tissue engineering has been applied for the regenerative medicine of several tissues including myocardial, corneal epithelial, esophageal, lung, liver, pancreatic, thyroidal, and periodontal tissue [21, 22, 24–31]. In some tissues, clinical trials have been started [25]. The application of cell sheet-based tissue engineering to myocardial tissue reconstruction is summarized in details in the following chapters.

## 3. The Fabrication of Electrically Communicative 3D Myocardial Tissue by Layering Cardiomyocyte Sheets In Vitro

Using a temperature-responsive culture surface, confluent neonatal rat cardiomyocytes can also be noninvasively harvested as a contiguous cell sheet simply by reducing the culture temperature (Figure 1) [24]. Because cell-cell junctions including gap junctions (GJs) between cardiomyocytes are conserved completely, the cardiomyocyte sheet can beat synchronously even just after detachment (electrograms in Figure 1) [32]. The establishment of electrical and functional couplings between layered cardiomyocyte sheets is a crucial point for the synchronous beatings of 3D myocardial tissue. Therefore, the electrical and functional interactions between layered cardiomyocyte sheets are analyzed precisely [32]. In vitro two cardiomyocyte sheets couple electrically at approximately 40 min after layering [32]. In addition, rapid GJ formation between layered cardiomyocyte sheets is also shown by a dye transfer assay and immunohistological analyses [32]. Furthermore, immunohistological analyses suggest the presence of cell surface GJ precursors on the cardiomyocyte sheet [32]. Because GJs are thought to be rapidly formed by only docking two GJ precursors on distinct two cell membranes [33], the preservation of GJ precursors on cardiomyocyte sheets must induce rapid electrical and functional couplings between layered cell sheets. In addition, deposited ECM on cardiomyocyte sheets also promotes the intimate attachment between layered cell sheets and may accelerate the docking of GJ precursors. These results show that in vitro complete electrically communicative 3D myocardial tissue can be fabricated by layering cardiomyocyte sheets. In fact, in vitro a multilayered cardiomyocyte sheet is known to beat spontaneously, synchronously, and macroscopically [24]. 

## 4. In Vivo Transplantation of Layered Cardiomyocyte Sheets

Next in vivo experiments using layered cardiomyocyte sheets are explained [24, 34, 35]. When layered cardiomyocyte sheets are transplanted into the subcutaneous tissue of nude rats, the transplanted grafts also pulsate synchronously and macroscopically, and interestingly surface electrograms originating from the grafts are able to be detectable independently from the host electrocardiograms [24, 34]. The histological analyses of implanted cardiomyocyte sheets show the characteristic structures of heart tissue including elongated cardiomyocytes, well-differentiated sarcomeres, GJs, and multiple blood vessels [35]. Long-term observation shows (1) the survival of beating grafts for more than one year and (2) the increase of their size, conduction velocity, and contractile force in proportion to the host growth, indicating the highly possible in vivo permanent survival of engineered myocardial tissues [34, 35]. The implanted cardiomyocyte sheets are found to be quite similar to real heart tissue. 

Cell sheets can be directly transplanted onto heart surface without suture, and the cells of sheets can be effectively delivered without cell loss [36]. In addition, after the transplantation of layered cardiomyocyte sheets onto infarcted rat heart, electrical and functional couplings between implanted myocardial tissues and host heart are established [36]. The pulsatile myocardial tissue grafts are expected to contribute the mechanical support of damaged heart via electrical and functional couplings.

## 5. The Therapeutic Effects of Cardiomyocyte Sheets Transplantation in Animal Models

Miyagawa et al. use rat damaged heart models to examine the therapeutic effects of cardiomyocyte sheet transplantation [37]. The transplantation of layered cardiomyocyte sheets into infarcted myocardium induces a significant increase in left ventricle (LV) wall thickness and a decrease in cross-sectional LV area [37]. In addition, cell sheet transplantation induces significant improvements in the LV ejection fraction (EF) and fractional shortening and a significant decrease in LV end-systolic area [37]. Furthermore, the transplantation of cardiomyocyte sheets induces the loss of branch block, which are likely to be related to fibrosis or necrosis in the heart tissue, in scar area [37]. These results indicate that (1) electrical connections are established between the implanted cardiomyocyte sheet and the host heart and (2) the cardiomyocyte sheet transplantation induces the restoration of damaged cardiac functions.

On the other hand, the importance of endothelial cell (EC) coculture within cardiomyocyte sheets on therapeutic effect is also reported [38]. In vitro, the cocultivation of ECs within cardiomyocyte sheets induces the expression of angiogenesis-related genes, namely, vascular endothelial growth factor (VEGF) and Cox-2 and the formation of EC-derived capillary-like prevascular network [39]. Using a temperature-responsive culture dish, these cell sheets including prevascular networks are able to be recovered and transplanted intactly [39]. Sekine et al. examine the therapeutic effects of prevascularized cardiomyocyte sheets and compare with those of EC-negative cardiomyocyte sheets using rat infarction model [38]. The transplantation of triple-layered cardiomyocyte sheet including EC networks induces the significant increase of blood-vessel densities in infarcted hearts in comparison to the transplantation of EC-negative layered cell sheet [38]. The improvements of the host heart functions are observed in proportion to the increase of EC numbers within cardiomyocyte sheets (The percent fractional shortening of the sham control group was 14 ± 4% (mean ± SD, *n* = 10); EC negative group: 19 ± 7%; 2 × 10^5^ EC transplantation group: 18 ± 4%; 4 × 10^5^ EC transplantation group: 22 ± 4%; 8 × 10^5^ EC transplantation group: 25 ± 5%) [38]. The transplantation of EC-positive cardiomyocyte sheets induces the significant reduction of fibrosis in the host damaged heart in comparison to the EC-negative cell sheets [38]. In vitro, EC-positive cardiomyocyte sheets produce a significantly greater amount of angiogenesis-related cytokines (basic fibroblast growth factor (bFGF), hepatocyte growth factor (HGF), and VEGF) in comparison to the EC-negative cell sheets [39]. VEGF and bFGF are strong promoters for angiogenesis, and HGF has an antiremodeling activity including antiapoptosis and antifibrosis in infarcted heart as well as angiogenesis [40–44]. Thus, the productions of these cytokines from implanted cell sheets including ECs are speculated to relate to the more effective improvements of damaged heart functions. 

## 6. Therapeutic Effects of Autologous Myoblast Sheets Transplantation in Animal Models

At present, clinical trials using human cardiomyocytes have been unaccomplished, though human embryonic stem (ES) cells [45] and induced pluripotent stem (iPS) cells [46, 47] have attractive potentials as pulsatile cardiomyocyte sources. On the other hand, autologous cells are used clinically for the therapy of cardiac disease as described above [4–10]. Myoblasts are used as the first cell source for the clinical trial of myocardial tissue repair [4]. Sawa and coworkers accomplish many investigations related to myoblast sheets [48–52]. Memon et al. examine the therapeutic effects of autologous skeletal myoblast sheets in rat infarction models and compare to those of myoblast injection [48]. The transplantation of myoblast sheets induces the significant improvement of LVEF and the shortening of the percentage of fractional area in comparison to the injection of myoblasts [48]. On the other hand, the myoblast injection also induces the improvement of the cardiac functions in comparison to the medium injection control [48]. The reduction in LV chamber area is observed in only cell sheet transplantation group [48]. Cell sheet transplantation induces a uniform and significantly thicker anterior wall, while there is no difference in the thickness between the cell injection and the control groups [48]. In addition, cell sheet transplantation induces the significant reduction of myocardial fibrosis in comparison to cell injection, which reduces fibrosis as compared to the control [48]. The RNA expression of stromal-derived factor 1 (SDF-1) and angiogenesis-related cytokines (HGF and VEGF) in myoblast sheet transplanted areas is significantly higher than that in myoblast injection areas [48]. SDF-1 recruits hematopoietic stem cells expressing CXCR4 [53, 54]. In fact, in infarcted heart, the recruitment of significant higher numbers of hematopoietic stem cells is observed in the myoblast sheet transplantation as compared to the myoblast injection and the control [48]. The production of SDF-1 as well as HGF and VEGF may also be related to the therapeutic effects of cell sheet transplantation. These data show that the cell sheet transplantation can induce more significant and remarkable improvement of cardiac functions than cell injection [48]. 

Memon et al. use a double-layered myoblast sheet in these experiments. Sekiya et al. analyze the effects of multilayered myoblast sheets to elucidate whether the increase of the number of cell sheets induces the improvement of cardiac function [51]. The transplantation of a quintuplet-layered myoblast sheet induces a significantly better improvement in heart functions (the reduction of heart hypertrophy, the inhibition of cardiac fibrosis, etc.) and a higher microvessel formation in the infarcted heart than a single-layered or a double-layered cell sheet [51]. The therapeutic effects of a triple-layered cell sheet are equal or somewhat smaller than those of a quintuplet-layered cell sheet [51]. In vitro, myoblast sheets promote the expression of angiogenesis factors in proportion to the number of cell sheets [51]. The transplantation of myoblast sheets also induces the organization of elastic fibers in infarcted heart via the expression of tropoelastin, which is expressed most strongly in a quintuplet-layered cell sheet [51]. The recovery of the elasticity of host heart via the reorganization of elastic fibers must also be related to the improvement of heart function. In conclusion, they describe that the improvement of cardiac function may plateau at a quintuplet-layered cell sheet [51]. 

In addition, experiments using middle or large animal models are performed [49, 50, 52]. The autologous transplantation of myoblast sheets also induces the restoration of heart with dilated cardiomyopathy by using a hamster model [49]. The transplantation of myoblast sheets improves a cardiac performance and prolongs the life-span of the animals, associating with the reorganization of the cytoskeletal proteins of host cardiac tissue and the reduction of myocardial fibrosis [49]. HGF induces not only the reduction of fibrosis but also the reorganization of cytoskeletal proteins such as alpha- and beta-sarcoglycans, which have a mechanical function to strengthen the plasma membrane during heart muscle contraction and an important role in the signal transduction of the tissue [55]. The reorganization of cytoskeletal proteins must be one cause of the improvement of cardiac function. Thus, the secretion of cytokines including HGF from implanted myoblast sheets may also be important for the improvement of cardiac functions in dilated cardiomyopathy. Hata et al. use a large animal dilated cardiomyopathy model, namely, a pacing-induced canine heart failure model [50]. Autologous myoblast sheet transplantation attenuates cardiac remodeling and improved LV systolic and diastolic function [50]. Miyagawa et al. use a porcine ischemic myocardium model [52]. Myoblast sheet transplantation induces the improvement of cardiac function by attenuating the cardiac remodeling in the porcine ischemic myocardium [52]. In rat and hamster models, small size cell sheets (diameter: approximately 10 mm) prepared on 35 mm temperature-responsive culture dishes are used [37, 38, 48, 49, 51]. On the other hand, in canine and porcine models, large size cell sheets (diameter: approximately 20–40 mm) prepared on 60 mm or 100 mm temperature-responsive culture dishes are used [50, 52]. Many cells can be effectively transplanted by the usage of large size cell sheets prepared on 100 mm dish. Based on these satisfactory results in several animal models, the clinical trial of autologous myoblast sheet transplantation is now in progress. In the clinical trials, large size cell sheets from 100 mm dishes are used. 

## 7. Therapeutic Effects of Adult Stem/Progenitor Cell Sheets Transplantation in Animal Models

The transplantations of several adult stem cell sheets (adipose-derived and menstrual blood-derived mesenchymal stem cell sheets and cardiac progenitor cell sheets) also give promising results in small animal models [56–58]. Miyahara et al. use rat adipose tissue-derived mesenchymal stem cell sheets [56]. In vitro, the stem cell sheets also produce the large amounts of HGF and VEGF [56]. The autologous transplantation of a single-layer adipose-tissue-derived stem cell sheet onto rat infarcted heart induces the growth of implanted cell sheet (the thickness: approximately 600 *μ*m) including many newly formed blood vessels [56]. The transplantation of the stem cell sheets induces the improvements of cardiac performances in damage heart with the reversal of cardiac wall thinning and the prolongation of survival after myocardial infarction [56]. On the other hand, in vivo cardiac differentiation from the implanted stem cells is scarcely observed [56]. Matsuura et al. use mouse cardiac progenitor cell sheets [57]. The autologous transplantation of the cardiac progenitor cell sheet induces the improvement of damaged heart function through the cardiomyocyte differentiations from the progenitor cells and paracrine effects mediated via the soluble vascular cell adhesion molecule 1 (VCAM-1)/very late antigen-4 (VLA-4) signaling pathway [57]. Hida et al. use human menstrual blood-derived mesenchymal stem cell sheets [58]. Interestingly, the human stem cells differentiate into spontaneous beating cardiomyocytes effectively by co-cultivating with mouse cardiomyocytes [58]. In addition, the transplantation of human menstrual blood-derived mesenchymal stem cell sheets also significantly restore damaged cardiac function, decreasing the myocardial infarction area in nude rat model [58]. Tissue including the stem-cell-derived cardiomyocytes is found in the implanted areas [58]. Bone-marrow-derived stem cell sheets are successfully fabricated, and their transplantations into large animal models are now in progress in several laboratories including ours. 

## 8. Future Possibilities of Myocardial Tissue Engineering

Generally, the therapeutic effects of transplanted tissue engineered grafts are mainly attributed to the following key factors:

1. mechanical support by transplanted pulsatile cardiomyocytes;
2. the secretion of several cytokines, including angiogenesis factors, from the transplanted tissues;
3. the formation of capillary networks at the site of myocardial infarction;
4. the inhibition of remodeling in damaged heart.

In relation to (1), the establishment of clinically applicable pulsatile cardiomyocyte source is important. In this point, ES/iPS cells are attractive, and the future progression of research is expected. In addition, the scale-up of pulsatile myocardial tissues is important. The fabrication and the transplantation of a large size cell sheet prepared on a 100 mm dish are succeeded. On the other hand, the insufficient supplies of oxygen and nutrients and waste accumulation limit their thickness and disturb the scale-up of tissue constructs. Our laboratory makes one solution for the problems by using the polysurgery method of cardiomyocyte sheets and fabricates a strongly pulsatile myocardial tissue having approximately 1 mm in thickness [59]. The trial of Hata et al. is also interesting [60]. They report the fabrication of a myocardial tissue with a thickness of approximately 800 *μ*m by combining cardiomyocyte sheets with cardiomyocytes-seeded decellularised porcine small-intestinal submucosa [60]. In relation to (2)–(4), several efforts, such as coculture with EC and the investigation of numbers of layered cell sheets, are performed as described in this paper. In addition, several possibilities to enhance the therapeutic effects of engineered tissue grafts for cardiac disease are discussed in many laboratories including ours [61, 62]. Although the myocardial tissue engineering including scaffold-free cell sheet-based tissue engineering stands at its start line, the technology is thought to have promising and enormous possibilities. 

## 9. Conclusions

Scaffold-free cell sheet-based tissue engineering is realized to be very useful for fabricating electrically communicative and pulsatile 3D myocardial tissue both in vitro and in vivo. The transplantation of myocardial tissue fabricated by cell sheet-based tissue engineering is a quite different cell delivery method from cell injection, and previous studies show promising and powerful potentials for curing damaged heart in several animal models. Cell sheet-based tissue engineering has promising and enormous potentials to cure many patients suffering from severe cardiac disease.

## References

[1] Mathers CD, Ezzati M, Lopez AD (2007). Measuring the burden of neglected tropical diseases: the global burden of disease framework. *PLoS Neglected Tropical Diseases*.

[2] The global burden of disease: 2004 update.

[3] Lloyd-Jones D, Adams RJ, Brown TM (2010). Executive summary: heart disease and stroke statistics—2010 update: a report from the American Heart Association. *Circulation*.

[4] Menasché P, Hagège AA, Scorsin M (2001). Myoblast transplantation for heart failure. *Lancet*.

[5] Menasché P, Alfieri O, Janssens S (2008). The myoblast autologous grafting in ischemic cardiomyopathy (MAGIC) trial: first randomized placebo-controlled study of myoblast transplantation. *Circulation*.

[6] Wollert KC (2008). Cell therapy for acute myocardial infarction. *Current Opinion in Pharmacology*.

[7] Jujo K, Ii M, Losordo DW (2008). Endothelial progenitor cells in neovascularization of infarcted myocardium. *Journal of Molecular and Cellular Cardiology*.

[8] Soejitno A, Wihandani DM, Kuswardhani RA (2010). Clinical applications of stem cell therapy for regenerating the heart. *Acta medica Indonesiana*.

[9] Alaiti MA, Ishikawa M, Costa MA (2010). Bone marrow and circulating stem/progenitor cells for regenerative cardiovascular therapy. *Translational Research*.

[10] Flynn A, O'Brien T (2011). Stem cell therapy for cardiac disease. *Expert Opinion on Biological Therapy*.

[11] Zhang M, Methot D, Poppa V, Fujio Y, Walsh K, Murry CE (2001). Cardiomyocyte grafting for cardiac repair: graft cell death and anti-death strategies. *Journal of Molecular and Cellular Cardiology*.

[12] Hofmann M, Wollert KC, Meyer GP (2005). Monitoring of bone marrow cell homing into the infarcted human myocardium. *Circulation*.

[13] Atala A, Lanza R, Thomson JA, Nerem R, Radisic M, Michael VM (2011). Cardiac tissue. *Principles of Regenerative Medicine*.

[14] Langer R, Vacanti JP (1993). Tissue engineering. *Science*.

[15] Li RK, Jia ZQ, Weisel RD, Mickle DAG, Choi A, Yau TM (1999). Survival and function of bioengineered cardiac grafts. *Circulation*.

[16] Leor J, Aboulafia-Etzion S, Dar A (2000). Bioengineered cardiac grafts: a new approach to repair the infarcted myocardium?. *Circulation*.

[17] Zimmermann WH, Melnychenko I, Wasmeier G (2006). Engineered heart tissue grafts improve systolic and diastolic function in infarcted rat hearts. *Nature Medicine*.

[18] Chachques JC, Trainini JC, Lago N (2007). Myocardial assistance by grafting a new bioartificial upgraded myocardium (MAGNUM clinical trial): one year follow-up. *Cell Transplantation*.

[19] Yamada N, Okano T, Sakai H (1990). Thermo-responsive polymeric surface: control of attachment and detachment of cultured cells. *Macromolecular Chemistry Rapid Communications*.

[20] Okano T, Yamada N, Sakai H, Sakurai Y (1993). A novel recovery system for cultured cells using plasma-treated polystyrene dishes grafted with poly(N-isopropylacrylamide). *Journal of Biomedical Materials Research*.

[21] Matsuda N, Shimizu T, Yamato M, Okano T (2007). Tissue engineering based on cell sheet technology. *Advanced Materials*.

[22] Masuda S, Shimizu T, Yamato M, Okano T (2008). Cell sheet engineering for heart tissue repair. *Advanced Drug Delivery Reviews*.

[23] Kushida A, Yamato M, Konno C, Kikuchi A, Sakurai Y, Okano T (1999). Decrease in culture temperature releases monolayer endothelial cell sheets together with deposited fibronectin matrix from temperature-responsive culture surfaces. *Journal of Biomedical Materials Research*.

[24] Shimizu T, Yamato M, Isoi Y (2002). Fabrication of pulsatile cardiac tissue grafts using a novel 3-dimensional cell sheet manipulation technique and temperature-responsive cell culture surfaces. *Circulation Research*.

[25] Nishida K, Yamato M, Hayashida Y (2004). Corneal reconstruction with tissue-engineered cell sheets composed of autologous oral mucosal epithelium. *New England Journal of Medicine*.

[26] Ohki T, Yamato M, Murakami D (2006). Treatment of oesophageal ulcerations using endoscopic transplantation of tissue-engineered autologous oral mucosal epithelial cell sheets in a canine model. *Gut*.

[27] Kanzaki M, Yamato M, Yang J (2007). Dynamic sealing of lung air leaks by the transplantation of tissue engineered cell sheets. *Biomaterials*.

[28] Ohashi K, Yokoyama T, Yamato M (2007). Engineering functional two- and three-dimensional liver systems in vivo using hepatic tissue sheets. *Nature Medicine*.

[29] Shimizu H, Ohashi K, Utoh R (2009). Bioengineering of a functional sheet of islet cells for the treatment of diabetes mellitus. *Biomaterials*.

[30] Arauchi A, Shimizu T, Yamato M, Obara T, Okano T (2009). Tissue-engineered thyroid cell sheet rescued hypothyroidism in rat models after receiving total thyroidectomy comparing with nontransplantation models. *Tissue Engineering—Part A*.

[31] Iwata T, Yamato M, Tsuchioka H (2009). Periodontal regeneration with multi-layered periodontal ligament-derived cell sheets in a canine model. *Biomaterials*.

[32] Haraguchi Y, Shimizu T, Yamato M, Kikuchi A, Okano T (2006). Electrical coupling of cardiomyocyte sheets occurs rapidly via functional gap junction formation. *Biomaterials*.

[33] Tadvalkar G, Pinto da Silva P (1983). In vitro, rapid assembly of gap junctions is induced by cytoskeleton disruptors. *Journal of Cell Biology*.

[34] Shimizu T, Yamato M, Kikuchi A, Okano T (2003). Cell sheet engineering for myocardial tissue reconstruction. *Biomaterials*.

[35] Shimizu T, Sekine H, Isoi Y, Yamato M, Kikuchi A, Okano T (2006). Long-term survival and growth of pulsatile myocardial tissue grafts engineered by the layering of cardiomyocyte sheets. *Tissue Engineering*.

[36] Sekine H, Shimizu T, Kosaka S, Kobayashi E, Okano T (2006). Cardiomyocyte bridging between hearts and bioengineered myocardial tissues with mesenchymal transition of mesothelial cells. *Journal of Heart and Lung Transplantation*.

[37] Miyagawa S, Sawa Y, Sakakida S (2005). Tissue cardiomyoplasty using bioengineered contractile cardiomyocyte sheets to repair damaged myocardium: their integration with recipient myocardium. *Transplantation*.

[38] Sekine H, Shimizu T, Hobo K (2008). Endothelial cell coculture within tissue-engineered cardiomyocyte sheets enhances neovascularization and improves cardiac function of ischemic hearts. *Circulation*.

[39] Sekiya S, Shimizu T, Yamato M, Kikuchi A, Okano T (2006). Bioengineered cardiac cell sheet grafts have intrinsic angiogenic potential. *Biochemical and Biophysical Research Communications*.

[40] Cittadini A, Grossman JD, Napoli R (1997). Growth hormone attenuates early left ventricular remodeling and improves cardiac function in rats with large myocardial infarction. *Journal of the American College of Cardiology*.

[41] Liu Y, Rajur K, Tolbert E, Dworkin LD (2000). Endogenous hepatocyte growth factor ameliorates chronic renal injury by activating matrix degradation pathways. *Kidney International*.

[42] Taniyama Y, Morishita R, Aoki M (2001). Therapeutic angiogenesis induced by human hepatocyte growth factor gene in rat and rabbit hindlimb ischemia models: preclinical study for treatment of peripheral arterial disease. *Gene Therapy*.

[43] Li Y, Takemura G, Kosai KI (2003). Postinfarction treatment with an adenoviral vector expressing hepatocyte growth factor relieves chronic left ventricular remodeling and dysfunction in mice. *Circulation*.

[44] Hinkel R, Trenkwalder T, Kupatt C (2011). Gene therapy for ischemic heart disease. *Expert Opinion on Biological Therapy*.

[45] Thomson JA (1998). Embryonic stem cell lines derived from human blastocysts. *Science*.

[46] Takahashi K, Tanabe K, Ohnuki M (2007). Induction of pluripotent stem cells from adult human fibroblasts by defined factors. *Cell*.

[47] Yu J, Vodyanik MA, Smuga-Otto K (2007). Induced pluripotent stem cell lines derived from human somatic cells. *Science*.

[48] Memon IA, Sawa Y, Fukushima N (2005). Repair of impaired myocardium by means of implantation of engineered autologous myoblast sheets. *Journal of Thoracic and Cardiovascular Surgery*.

[49] Kondoh H, Sawa Y, Miyagawa S (2006). Longer preservation of cardiac performance by sheet-shaped myoblast implantation in dilated cardiomyopathic hamsters. *Cardiovascular Research*.

[50] Hata H, Matsumiya G, Miyagawa S (2006). Grafted skeletal myoblast sheets attenuate myocardial remodeling in pacing-induced canine heart failure model. *Journal of Thoracic and Cardiovascular Surgery*.

[51] Sekiya N, Matsumiya G, Miyagawa S (2009). Layered implantation of myoblast sheets attenuates adverse cardiac remodeling of the infarcted heart. *Journal of Thoracic and Cardiovascular Surgery*.

[52] Miyagawa S, Saito A, Sakaguchi T (2010). Impaired myocardium regeneration with skeletal cell sheets-A preclinical trial for tissue-engineered regeneration therapy. *Transplantation*.

[53] Askari AT, Unzek S, Popovic ZB (2003). Effect of stromal-cell-derived factor 1 on stem-cell homing and tissue regeneration in ischaemic cardiomyopathy. *Lancet*.

[54] Yamaguchi JI, Kusano KF, Masuo O (2003). Stromal cell-derived factor-1 effects on ex vivo expanded endothelial progenitor cell recruitment for ischemic neovascularization. *Circulation*.

[55] Kondoh H, Sawa Y, Fukushima N (2005). Reorganization of cytoskeletal proteins and prolonged life expectancy caused by hepatocyte growth factor in a hamster model of late-phase dilated cardiomyopathy. *Journal of Thoracic and Cardiovascular Surgery*.

[56] Miyahara Y, Nagaya N, Kataoka M (2006). Monolayered mesenchymal stem cells repair scarred myocardium after myocardial infarction. *Nature Medicine*.

[57] Matsuura K, Honda A, Nagai T (2009). Transplantation of cardiac progenitor cells ameliorates cardiac dysfunction after myocardial infarction in mice. *Journal of Clinical Investigation*.

[58] Hida N, Nishiyama N, Miyoshi S (2008). Novel cardiac precursor-like cells from human menstrual blood-derived mesenchymal cells. *Stem Cells*.

[59] Shimizu T, Sekine H, Yang J (2006). Polysurgery of cell sheet grafts overcomes diffusion limits to produce thick, vascularized myocardial tissues. *FASEB Journal*.

[60] Hata H, Bär A, Dorfman S (2010). Engineering a novel three-dimensional contractile myocardial patch with cell sheets and decellularised matrix. *European Journal of Cardio-thoracic Surgery*.

[61] Bonaros N, Rauf R, Schachner T, Laufer G, Kocher A (2008). Enhanced cell therapy for ischemic heart disease. *Transplantation*.

[62] Kobayashi H, Shimizu T, Yamato M (2008). Fibroblast sheets co-cultured with endothelial progenitor cells improve cardiac function of infarcted hearts. *Journal of Artificial Organs*.

